# Post-exercise Supplementation of Sodium Bicarbonate Improves Acid Base Balance Recovery and Subsequent High-Intensity Boxing Specific Performance

**DOI:** 10.3389/fnut.2019.00155

**Published:** 2019-10-01

**Authors:** Lewis A. Gough, Steven Rimmer, S. Andy Sparks, Lars R. McNaughton, Matthew F. Higgins

**Affiliations:** ^1^Department of Sport and Exercise, Research Centre for Life and Sport Sciences (CLaSS), School of Health Sciences, Birmingham City University, Birmingham, United Kingdom; ^2^Sports Nutrition and Performance Group, Department of Sport and Physical Activity, Edge Hill University, Ormskirk, United Kingdom; ^3^Human Sciences Research Centre, University of Derby, Derby, United Kingdom; ^4^Department of Sport and Movement Studies, Faculty of Health Science, University of Johannesburg, Johannesburg, South Africa

**Keywords:** buffering, alkalosis, acid base balance, combat sports, recovery, nutrition, training

## Abstract

The aim of this study was to assess the effects of post-exercise sodium bicarbonate (NaHCO_3_) ingestion (0.3 g.kg^−1^ body mass) on the recovery of acid-base balance (pH, ${\text{HCO}}_{3}^{-}$, and the SID) and subsequent exercise performance in elite boxers. Seven elite male professional boxers performed an initial bout of exhaustive exercise comprising of a boxing specific high-intensity interval running (HIIR) protocol, followed by a high-intensity run to volitional exhaustion (T_LIM1_). A 75 min passive recovery then ensued, whereby after 10 min recovery, participants ingested either 0.3 g.kg^−1^ body mass NaHCO_3_, or 0.1 g.kg^−1^ body mass sodium chloride (PLA). Solutions were taste matched and administered double-blind. Participants then completed a boxing specific punch combination protocol, followed by a second high-intensity run to volitional exhaustion (T_LIM2_). Both initial bouts of T_LIM1_ were well matched between PLA and NaHCO_3_ (ICC; *r* = 0.94, *p* = 0.002). The change in performance from T_LIM1_ to T_LIM2_ was greater following NaHCO_3_ compared to PLA (+164 ± 90 vs. +73 ± 78 sec; *p* = 0.02, CI = 45.1, 428.8, *g* = 1.0). Following ingestion of NaHCO_3_, pH was greater prior to T_LIM2_ by 0.11 ± 0.02 units (1.4%) (*p* < 0.001, CI = 0.09, 0.13, *g* = 3.4), whilst ${\text{HCO}}_{3}^{-}$ was greater by 8.8 ± 1.5 mmol.l^−1^ (26.3%) compared to PLA (*p* < 0.001, CI = 7.3, 10.2, *g* = 5.1). The current study suggests that these significant increases in acid base balance during post-exercise recovery facilitated the improvement in the subsequent bout of exercise. Future research should continue to explore the role of NaHCO_3_ supplementation as a recovery aid in boxing and other combat sports.

## Introduction

High levels of glycolytic flux are essential to maintain the required physiological output during combat exercise (1), although a concomitant fall in both muscle and blood pH and bicarbonate ion concentration [${\text{HCO}}_{3}^{-}$] eventually occurs (2). This is due to the increases in hydrogen ion (H^+^) accumulation, which in turn, disturb the state of equilibrium between acidity and alkalinity of body fluids (i.e., acid base balance). Such an alteration is known as metabolic acidosis and has been associated with fatigue by reducing or impairing the release of calcium ions (Ca^2+^) from the sarcoplasmic reticulum (3), impeding glycolytic enzyme activity (4), and altering the strong ion difference leading to reduced action potentials and muscle excitability (5). The typical daily regimen for a competitive boxer often consists of two sessions comprised of an initial high-intensity intermittent running session followed by a boxing-specific session that mimics the demands of competition, interspersed within a short recovery period (6). Subsequently a large degree of metabolic acidosis is likely evident in the subsequent bout of exercise, therefore mitigation of the deleterious effects between sessions are prudent to investigate.

Pre-exercise ingestion of 0.3 g.kg^−1^ body mass (BM) sodium bicarbonate (NaHCO_3_) can lead to an approximate increase in pH (+0.07 ± 0.01) and ${\text{HCO}}_{3}^{-}$ from baseline (+3.9 ± 0.9 mmol^.^l^−1^), eliciting a state of metabolic alkalosis (7). Ergogenic effects have been reported in combat sports including boxing (8) and judo (9, 10) by either increasing punches landed or total work done (TWD). Whilst the effects of pre-exercise NaHCO_3_ ingestion has been well-researched [for review see (11)], the effects of post-exercise ingestion between two bouts of exercise to promote recovery has received minimal attention. The use of this alternative method might permit a greater observed improvement in acid base balance during the recovery period, whilst the enhanced level of acid base balance would not have been utilized within the initial bout of exercise. These factors combined might therefore increase performance during the subsequent bout of exercise compared to pre-exercise NaHCO_3_ ingestion. Indeed, Gough et al. (12) reported that 0.3 g.kg^−1^ NaHCO_3_ ingested 30 min into a 90 min post-exercise recovery period improved subsequent cycling time to volitional exhaustion by 33 s (~14%) in recreationally active individuals. It is likely that an enhanced level of acid base balance was the primary mechanism for such an improvement, as the authors reported marked increases in pH and ${\text{HCO}}_{3}^{-}$ prior to the second bout of exercise compared to the placebo [pH = +0.07, effect size (ES) = 2.6, ${\text{HCO}}_{3}^{-}$ = +7 mmol^.^l^−1^, ES = 3.4]. It is unknown, however, if these positive findings translate to other exercise modalities such as boxing, and individuals of a higher training status.

The mechanisms to explain the performance improvement following NaHCO_3_ supplementation is not unique to changes in pH and ${\text{HCO}}_{3}^{-}$. Specifically, marked ionic shifts are suggested to contribute to muscle fatigue by impeding maximal Na^+^, K^+^-ATPase activity, subsequently impairing cell membrane excitability (2, 5, 13). Indeed, both large effluxes of extracellular K^+^ concomitant with reductions in Na^+^ have been suggested to exacerbate the K^+^ induced decline in force production (14). Pre-exercise ingestion of NaHCO_3_ has been shown to reduce K^+^ and increase Na^+^ prior to the onset of exercise (5, 8, 13, 15). Indeed, Siegler and Hirscher (8) reported NaHCO_3_ supplementation prior to a simulated boxing protocol lowered K^+^ compared to the placebo condition (4.0 ± 0.1 mEq.l^−1^ vs. 5.3 ± 0.4 mEq.l^−1^, respectively) and subsequently speculated that this reduction might have facilitated the resulting performance improvement. It is widely argued however, that electrolyte balance should be assessed by the collective analysis of the strong ion difference (SID), which is the balance of the fully dissociated cations and anions in intracellular and extracellular fluid (16). Synergistic changes in electrolytes are suggested to allow for deeper assessment of fatigue mechanisms, as opposed to reporting changes within a single electrolyte. In the only study to date, Gough et al. (17) reported a significant increase in the SID following NaHCO_3_ supplementation and an improvement in 2 × 4 km time trial cycling bouts interspersed by 40 min recovery, although this study was conducted in a normobaric hypoxic environment. The purpose of this study therefore was to investigate the effects of post-exercise ingestion of NaHCO_3_ on acid base balance recovery, the SID and subsequent boxing performance.

## Materials and Methods

Seven male elite professional boxers [age: 27.1 ± 5.1 years, stature: 175.8 ± 5.7 cm, body mass: 72.2 ± 10.3 kg, relative peak oxygen uptake (VO_2peak_): 55.8 ± 11.4 ml·kg·min^−1^] from various boxing weight classifications including flyweight, lightweight, junior welterweight (WBO/IBF) super lightweight (WBA/WBC), middleweight, and super middleweight completed this study. Participants were considered elite standard boxers and were at least Commonwealth (British Empire), English, International Masters, British Masters, or Midlands Area title holders, with an average of 4.1 ± 3.6 years professional boxing experience. At the time of data collection, all participants were in pre-competition training. The study received institutional ethics committee approval (University of Derby, UK) prior to any testing, and participants were informed of the details of the study, both verbally and in writing, prior to providing written informed consent in accordance with the Declaration of Helsinki. Physical Activity Readiness Questionnaire (PAR-Q) and blood analysis questionnaires were completed prior to each bout of exercise.

### Preliminary Procedures

Prior to each trial, participants were requested to avoid strenuous exercise and to abstain from caffeine and alcohol ingestion for at least 24 h. Participants were also encouraged to adopt the same mixed balanced diet with adherence monitored through a food diary, which participants recorded 24 h prior to testing. A photocopy of the food diary was given to each participant to facilitate dietary replication prior to each experimental trial with 100% adherence achieved. Finally, participants were verbally screened to ensure they had refrained from ingestion of ergogenic buffers such as sodium citrate and β-alanine for 6 months prior to beginning the study.

Participant's body composition was assessed using Dual Energy X-ray Absorptiometry (Lunar iDXA, GE Healthcare, Hertfordshire, UK) 7–10 days prior to the experimental trials for analysis of body mass (kg). During the same visit, following 3 h of fasting, participants completed an incremental exercise test on a motorized treadmill (Desmo, Woodway, Germany) to assess peak oxygen uptake (VO_2peak_). Initially participants warmed up for 5 min at 8 km·h^−1^ with a 0% gradient. The test began with a 3 min stage at 10 km·h^−1^, subsequently the speed increased by 2 km·h^−1^ every three min until it reached the 16 km·h^−1^ stage. From this point the gradient was increased by 2% every 2 min until volitional exhaustion. Throughout the test expired gas samples were collected via an online breath by breath system (Cortex MetaLyzer II, Biophysik, Leipzig, Germany) which was calibrated before each test as per the manufacturer's guidelines. Expired gas samples were analyzed for oxygen consumption (VO_2_), carbon dioxide production (VCO_2_) and respiratory exchange ratio (RER). The highest value of VO_2_ obtained in any 30 s period was used to calculate VO_2peak_.

### Familiarization

During the second laboratory visit participants were familiarized with the high intensity interval run (HIIR) protocol (Table 1), and the punch type techniques and combinations (Table 2), that would be utilized during experimental trials. In the HIIR, emphasis was placed upon exercising at a percentage of running velocity at VO_2peak_ during each differing work interval as opposed to heart rate ensuring the total time at each workload was readily matched. Finally, participants ran at a velocity that elicited 90% VO_2peak_ to volitional exhaustion (T_LIM_) as a measure of high-intensity endurance capacity.

**Table 1:** Example of one round of the high intensity interval run (HIIR) protocol.

**Exercise duration (sec)**	****~**%VO_**2*PEAK***_**	**Intensity level**
30	90	High
30	75	Moderate
30	90	High
30	75	Moderate
30	90	High
30	75	Moderate
30	90	High
30	75	Moderate
60 AR^*^	SS^**^	Low

AR* *active recovery*;

SS** *self-selected during familiarization*.

**Table 2:** Punch combinations sequence utilized during boxing specific performance.

**Phase 1**	**Phase 2 (combinations)**	**Phase 3**
MIR	J-	MOR
MIR	J-BH	MOR
MIR	J-BH-LU	MOR
MIR	J-BH-LU-BU	MOR
MIR	J-BH-LU-BU-LH	MOR
MIR	J-BH-LU-BU-LH- BHH	MOR

*MIR, move in range; MOR, move out of range; J, Jab; BH, backhand; LU, lead uppercut; BU, backhand uppercut; LH, lead hook; BHH, backhand hook*.

### Experimental Design and Protocol

Experimental trials were conducted using a repeated measures, partially counterbalanced (due to odd number sample size), double-blind, and placebo controlled design, each separated by 7 days. Participants reported for each trial 3 h postprandial and at the same time of day to avoid any circadian rhythm effects on performance (18). Body mass was measured and recorded at the start of each laboratory visit (Seca 761 weight scales, Birmingham, UK), to monitor possible fluctuations between experimental trials due to the participants being in pre-competition training stages. The following baseline measures were obtained after 5 min seated rest: heart rate (HR); (Polar, FT40, Finland), blood lactate concentration [Bla^−^], base excess (BE), bicarbonate ion concentration [${\text{HCO}}_{3}^{-}$] and a range of electrolytes (sodium [Na^+^], potassium [K^+^], calcium [Ca^2+^], and chloride [Cl^−^]). The electrolyte data was used to calculate the apparent SID using an online spreadsheet (19) based on the following formula: [K^+^] + [Na^+^] + [Ca^2+^] + [Na^+^] – [Cl^−^] – [Bla^−^]. Blood variables were collected via a finger prick capillary blood sample and analyzed with a blood gas analyser (ABL90 Flex, Radiometer, West Sussex, UK). Perceived readiness to exercise (PRE) was then recorded against an 11 point (0–10) scale with 0 representing “not at all ready to exercise” and 10 representing “completely ready to exercise” (20).

Exercise trials commenced with a 5 min treadmill run at a velocity eliciting ~60% VO_2PEAK_ (warm-up) immediately followed by the HIIR protocol (Table 1) which was repeated three times to imitate the demands of 3 × 4 min boxing rounds, each separated by 60 s active recovery. A self-selected active recovery was recorded and replicated for each recovery interval in both experimental trials. Subsequently, a fourth and final bout was performed on a treadmill at a running velocity eliciting ~90% VO_2PEAK_ to volitional exhaustion (T_LIM1_) with participants blinded from distance and time completed. Overall (i.e., related to cardiovascular strain) ratings of perceived exertion (RPE_O_) (21) were recorded within the final 5 s of each round. Immediately post-exercise HR and RPE_O_ were recorded. Five min post-exercise HR and blood metabolite/electrolyte data was collected as previously described.

Participants then recovered passively for 75 min prior to undertaking subsequent boxing performance. This was selected due to previous data showing this time period is approximately when acid base balance returns to baseline following high-intensity exercise (12). Ten minutes into recovery, participants consumed either 0.3 g.kg^−1^ body mass of NaHCO_3_ or 0.1 g.kg^−1^ body mass of sodium chloride (placebo; PLA) within a standardized 5 min period. This time period was selected due to the fear of vomiting if ingestion began immediately post-exercise, whilst a longer time period was not used as this may have allowed acid base balance to recover back to baseline values prior to ingestion (12). Both drinks were mixed in 4 ml·kg^−1^ body mass tap water and 1 ml.kg^−1^ body mass of double strength no added sugar orange squash (Sainsbury's, London, UK) (20). Thirty minutes post exercise abdominal discomfort (AD) and gut fullness (GF) were recorded using an 11 point (0–10) scale, with 0 representing “empty” and “completely comfortable,” and 10 representing “bloated” and “unbearable pain,” respectively (20). Water was consumed *ad libitum* during recovery (mean 582 ± 40 ml).

At the end of the 75 min recovery, HR, PRE, blood metabolites/electrolytes, AD, and GF were all recorded prior to participants performing a 5 min standardized dynamic warm up. Participants then completed the boxing specific protocol (Table 2) whereby they were required to strike the focus pads (Serious, Rapid Fire Punch Mitts, London, UK), which were worn by the same researcher for all trials. Each complete cycle consisted of 21 punches with participants instructed to stay in their preferred boxing stance (orthodox or southpaw) throughout. The punch combination cycle was performed repeatedly for 3 × 3 min rounds, each separated by 60 s passive recovery. Participants were all given the same boxing gloves (10 oz, Adidas, Hi-Tech Multi-Boxing Glove, Germany) for both experimental trials. An audio and visual boxing gym timer (Title Boxing, De luxe gym timer, USA) kept timing of rounds. Immediately at the end of each round participants HR, RPE_O_ and ratings of perceived exertion localized to the arms (RPE_A_; Borg scale 6–20) were recorded. Upon completion of the 3 boxing specific rounds AD and GF were also recorded. Following a 60 s rest period, participants then performed a final high intensity treadmill run corresponding to a speed that elicited ~90% VO_2peak_ to volitional exhaustion (T_LIM2_). Immediately post exercise HR, RPE_O_, AD, and GF were recorded, and 5 min post-exercise HR, blood metabolite/electrolytes, AD, and GF were recorded.

### Statistical Analysis

Data was firstly checked for normality via a Shaprio-Wilk test, followed by a Mauchly test for homogeneity of variance/sphericity. A paired *t*-test was used for some performance (T_LIM1_ and T_LIM2_) and blood/perceptual data (change in ${\text{HCO}}_{3}^{-}$ during T_LIM2_, change in ${\text{HCO}}_{3}^{-}$ during recovery, and aggregated GI discomfort). A two-way [treatment × time] repeated measures ANOVA was conducted with a Bonferroni correction for changes in blood variables (pH, ${\text{HCO}}_{3}^{-}$, and lactate). Effect size (ES) for interactions from the ANOVA are reported as partial eta squared (Pη^2^), whilst between treatment ES are reported as Hedge's g effect sizes (*g*) [interpreted as per conventional thresholds described by (22)]. If *p* < 0.05 then 95% CI are reported, where changes that do not cross the zero boundary treated as significant. A Friedman test was used for non-normally distributed data (AD, GF), and where the a priori alpha value was observed (i.e., *p* < 0.05) a *post hoc* Wilcoxon signed rank-test was conducted with median, *z* score, and *p*-value reported. For non-normally distributed data the ES is calculated by Z/√ n with 0.10, 0.24, and 0.37 considered as small, medium, and large effects, respectively (23). Reproducibility of the performance in T_LIM1_ was assessed using intraclass correlation coefficients (ICC), with the *r*-value and significance reported. Additional statistics such as confidence intervals and effect sizes were used due to the small sample size in the study, which might not be suited to statistical procedures such as *t*-test and ANOVA in isolation. Data were analyzed using a statistical software package, SPSS (V.24, IBM Inc., Chicago, IL, USA).

## Results

### Performance

**Figure 1:**
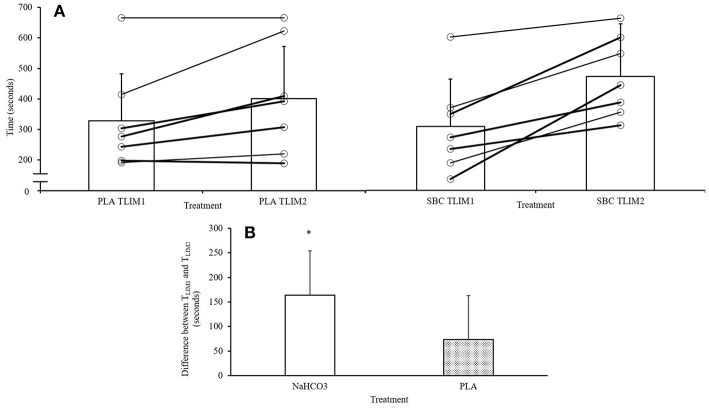
Overview of performance responses following NaHCO_3_ or PLA. *NaHCO_3_ greater than PLA (*p* < 0.05). **(A)** Changes between T_LIM1_ and T_LIM2_, **(B)** change in performance from T_LIM1_ and T_LIM2_ following NaHCO_3_ or Placebo.

Both initial bouts for T_LIM1_ were well-matched between PLA and NaHCO_3_ (328 ± 155 vs. 307 ± 142 s; ICC: *r* = 0.94, *p* = 0.002; *t*-test, *p* = 0.526), showing that participants were at a similar level of fatigue at the start of the recovery period. Performance in T_LIM2_ was greater by 70 ± 90 s (28%) following NaHCO_3_ compared to PLA (*p* = 0.084, CI = −153.8, 12.9; Figure 1A), with a moderate effect size (*g* = 0.41). The change in performance from T_LIM1_ to T_LIM2_ was greater following NaHCO_3_ compared to PLA (+164 ± 90 vs. +73 ± 78 sec; *p* = 0.02, CI = 45.1, 428.8, *g* = 1.0; Figure 1B). One participant displayed an ergolytic effect following NaHCO_3_ ingestion, such that T_LIM2_ decreased by 13% compared to PLA (545 vs. 623 s). This participant also suffered from moderate to severe GI discomfort.

### Blood Variables

**Figure 2:**
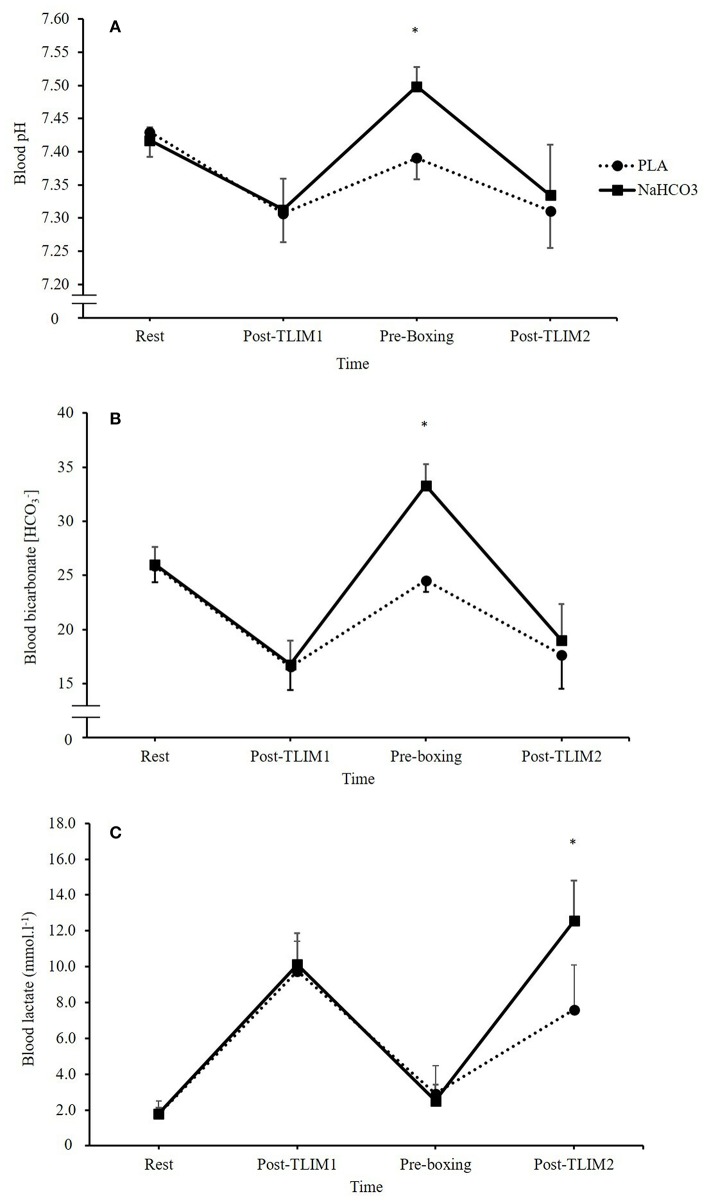
Blood acid base balance responses following NaHCO_3_ or PLA, where **(A)** pH, **(B)** blood bicarbonate [${\text{HCO}}_{3}^{-}$], and **(C)** blood lactate [BLa^−^]. *NaHCO_3_ greater than PLA (*p* < 0.05).

No differences in pH between PLA and NaHCO_3_ were observed at baseline (7.43 ± 0.04 vs. 7.42 ± 0.02; *p* = 0.233), or post T_LIM1_ (7.31 ± 0.04 vs. 7.31 ± 0.04; *p* = 0.696). Following the recovery period, and the ingestion of NaHCO_3_, pH was greater prior to T_LIM2_ by 0.11 ± 0.02 units (1.4%) (*p* < 0.001, CI = 0.09, 0.13, *g* = 3.4). Post T_LIM2_, no difference between treatments was observed for pH (7.31 ± 0.06 vs. 7.33 ± 0.08; *p* = 0.271; Figure 2A). There were no differences in ${\text{HCO}}_{3}^{-}$ between PLA and NaHCO_3_ at baseline (25.9 ± 1.5 vs. 26.0 ± 1.6 mmol.l^−1^; *p* = 0.750), post T_LIM1_ (16.6 ± 2.2 vs. 16.8 ± 2.2 mmol.l^−1^; *p* = 0.723), or post T_LIM2_ (17.7 ± 3.1 vs. 19.0 ± 3.4 mmol.l^−1^; *p* = 0.196). Following recovery however, ${\text{HCO}}_{3}^{-}$ was greater by 8.8 ± 1.5 mmol.l^−1^ (26.3%) post-NaHCO_3_ supplementation compared to PLA (*p* < 0.001, CI = 7.3, 10.2, *g* = 5.1; Figure 2B). The change in ${\text{HCO}}_{3}^{-}$ during recovery (post T_LIM1_ to pre T_LIM2_) was greater following NaHCO_3_ ingestion compared to PLA (16.6 ± 1.4 vs. 8.0 ± 2.1 mmol.l^−1^; *p* < 0.001; CI = 6.5, 10.7, *g* = 4.5). During T_LIM2_, the change in ${\text{HCO}}_{3}^{-}$ during exercise was greater for NaHCO_3_ compared to PLA (14.3 ± 2.9 vs. 6.9 ± 2.5 mmol.l^−1^ *p* < 0.001, 10.3, 4.5, *g* = 2.5). Post T_LIM2_, BLa^−^ was 5.2 ± 2.6 mmol.l^−1^ (39.5%) greater following NaHCO_3_ (*p* = 0.002, CI = 2.6, 7.3, *g* = 2.0), with no difference at any other time point (*p* > 0.05; Figure 2C).

**Figure 3:**
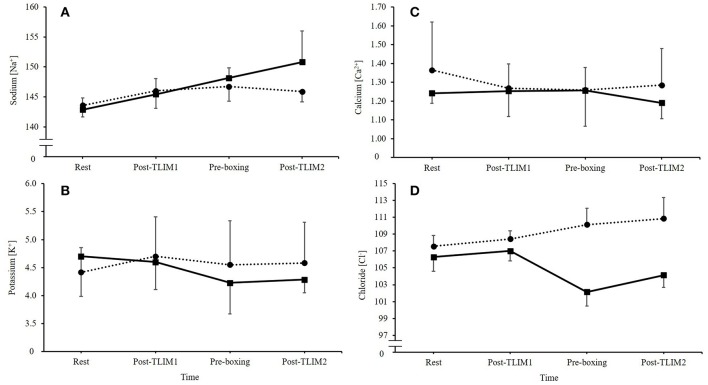
Changes in extracellular electrolytes following NaHCO_3_ or PLA, where **(A)** sodium [Na^+^], **(B)** potassium [K^+^], **(C)** calcium [Ca^2+^], and **(D)** chloride [Cl^−^].

**Figure 4:**
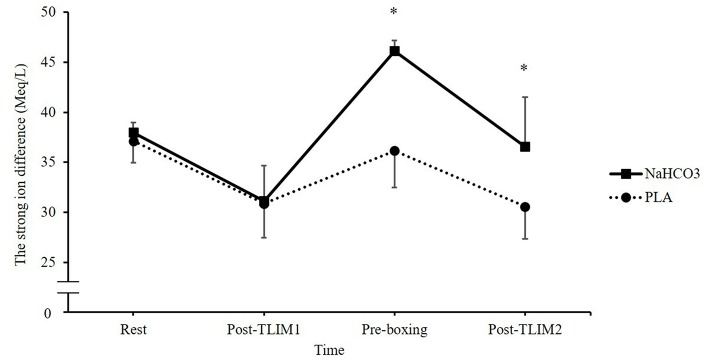
Changes in blood strong ion difference (SID) following NaHCO_3_ or PLA. *NaHCO_3_ greater than PLA (*p* < 0.05).

Ingestion of NaHCO_3_ caused marked changes in Na^+^, K^+^, Ca^2+^, and Cl^−^ (Figure 3). A time^*^treatment interaction was observed for the SID (*p* = 0.023, Pη^2^ = 0.576), such that a 10% increase in the SID was observed post recovery following NaHCO_3_ ingestion compared to PLA (46 ± 1 vs. 36 ± 4 meq/l; *p* < 0.001, CI = 6.3, 13.7, *g* = 3.2; Figure 4).

### Heart Rate and Perceptual Measures

Post-exercise ingestion of NaHCO_3_ increased HR in rounds 2 and 3 compared to PLA (*p* < 0.05), whilst no effect was observed on RPE_O_ or RPE_A_ (*p* > 0.05) during any round of the HIIR. No effect on post T_LIM2_ HR (*p* = 0.217, *g* = 0.46) was observed following NaHCO_3_. Likewise, no difference in HR between NaHCO_3_ and PLA were observed at any time point during T_LIM2_ (all *p* > 0.05). Similarly, NaHCO_3_ supplementation had no effect on post T_LIM2_ RPE_O_ (*Z* = 1.47, *p* = 0.383), with no difference observed between treatments at any time point (*p* > 0.05).

**Figure 5:**
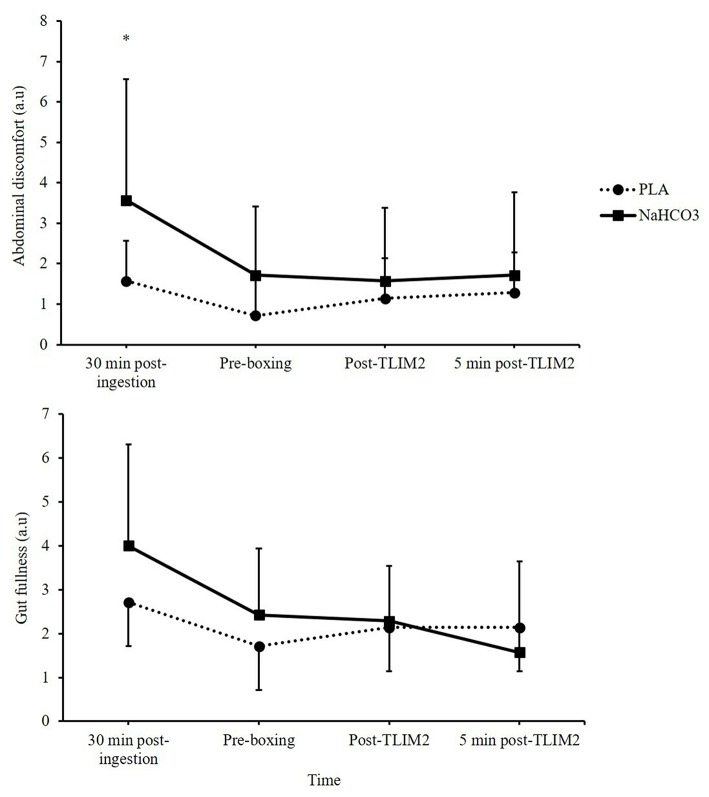
Gastrointestinal (GI) discomfort (gut fullness and abdominal discomfort) following NaHCO_3_ or PLA. *NaHCO_3_ greater than PLA (*p* < 0.05).

Abdominal discomfort was greater following NaHCO_3_ ingestion at 30 min recovery, displaying a moderate effect size (3.6 ± 3.0 vs. 1.6 ± 2.3; *Z* = 1.76, *p* = 0.07, *g* = 0.7). At the end of recovery, abdominal discomfort had generally reduced, although NaHCO_3_ was still greater (1.7 ± 1.7 vs. 0.7 ± 1.3; *Z* = −1.89, *p* = 0.06, *g* = 0.6). No time^*^treatment interaction was observed for gut fullness (*p* = 0.219, η^2^ = 0.213). Aggregated GI discomfort was not significantly different between NaHCO_3_ ingestion and PLA (19 ± 13 vs. 13 ± 15; *p* = 0.175, −14.1, 3.2), although it was associated with a moderate effect size (*g* = 0.40; Figure 5).

## Discussion

This study investigated the effects of post-exercise NaHCO_3_ ingestion on subsequent high-intensity boxing performance. Following NaHCO_3_ ingestion, acid base balance was increased prior to T_LIM2_ compared to PLA which subsequently improved subsequent boxing specific exercise performance. Athletes and coaches can therefore implement this strategy to support training at times when multiple bouts of exercise are carried out with limited recovery interspersed.

The findings of the current study show that NaHCO_3_ ingestion improved subsequent boxing specific performance, by markedly reducing the decline from T_LIM1_ to T_LIM2_. This adds to previous work evaluating post-exercise NaHCO_3_ supplementation as a recovery supplement (12). Indeed, Gough et al. (12) showed that NaHCO_3_ ingestion 30 min into a 90 min recovery period improved subsequent cycling capacity, such that a moderate effect size (*g* = 0.5) was observed vs. the placebo within a group of recreationally trained males. The current study adds however, similar ergogenic effects can be achieved with post-exercise NaHCO_3_ ingestion within a shorter recovery time, individuals of a higher training status, and combat exercise. In addition, these findings also support previous literature showing NaHCO_3_ ingestion is an effective supplement to improve combat performance when ingested prior to exercise (8, 24). Future research could consider the impact of NaHCO_3_ ingestion to enhance subsequent performance in other combat sports.

Based on the observed improvements it can be speculated that if NaHCO_3_ supplementation could be adapted into a chronic weekly supplementation strategy, this might lead to greater adaptation to training. Previous work by Percival et al. (25) has shown mRNA expression of PGC-1a, a known mechanism for mitochondrial adaptation, was increased 3 h following a high intensity training session with acute NaHCO_3_ ingestion compared to a placebo. Based on this evidence it is plausible that this may aid training adaptation in boxing, however, the study by Percival et al. (25) was in cycling and in lesser trained individuals to the current study (healthy men vs. elite boxers). In addition, other studies investigating the effects of NaHCO_3_ ingestion to support training adaptations are equivocal within trained individuals. Indeed, Edge et al. (26) reported chronic NaHCO_3_ ingestion significantly increased lactate threshold by 11% and time to fatigue (100% VO_2peak_) by 41% compared to a placebo following 8 weeks of cycling interval training. Both Driller et al. (27) and Siegler et al. (28) however, have shown no greater training adaptations following NaHCO_3_ ingestion within rowing and resistance exercise modalities across 4 and 10 weeks of training, respectively. Considering positive findings have been reported in combat exercise following NaHCO_3_ ingestion (8, 24), further research could explore if greater training adaptations occur with chronic NaHCO_3_ ingestion.

In the present study, the likely mechanism to explain the improvement in subsequent performance is the changes in blood acid base balance between bouts, such that pH, ${\text{HCO}}_{3}^{-}$, and the SID were significantly higher at 75 min recovery following NaHCO_3_ ingestion. Full recovery of pH, ${\text{HCO}}_{3}^{-}$, and the SID was achieved in approximately 30–35 min. This is in contrast to the placebo condition, which failed to recover any of these blood analytes to baseline within 75 min of recovery. As a result, NaHCO_3_ ingestion mitigated the disturbance to acid base balance during T_LIM2_, which subsequently may explain the performance improvement. Such a greater state of metabolic alkalosis has been shown to increase buffering capacity by facilitating efflux of H^+^ from the active muscle by enhanced circulating ${\text{HCO}}_{3}^{-}$, and thus, increasing the glycolytic energy contribution to high-intensity exercise (24, 29). The current study supports these mechanisms, reporting a 2-fold increase in the ${\text{HCO}}_{3}^{-}$ change during T_LIM2_, and a marked increase in lactate post-T_LIM2_ following NaHCO_3_ ingestion. Indeed, Lopes-Silva et al. (24) showed similar changes in post-exercise lactate following NaHCO_3_ ingestion, but also reported a significant 31% increase in estimated glycolytic activity during simulated taekwondo combat. It is important to note however, the link between metabolic acidosis and fatigue has been widely criticized, suggesting at physiologically valid muscle temperatures, accumulation of H^+^ has limited effects on muscle contractile ability (30). As the current study did not assess either temperature or metabolite accumulation in muscle, we cannot confirm that acidosis has a direct impact on fatigue and performance.

An alternative mechanism to explain the performance improvement might be the increases in the SID following NaHCO_3_ ingestion. Reductions in K^+^ and Cl^−^ were observed, whilst Na^+^ was increased in the recovery period, which lead to an overall increase in the SID. This could lead to an increase in electrical excitation, membrane potentials and muscle action potentials, which in turn, could support maximal Na^+^, K^+^-ATPase activity (2, 31). Previous research, however, has suggested the most important electrolyte change is K^+^, by demonstrating that raised extracellular concentration depresses muscle excitability (31). This suggests that the important changes that NaHCO_3_ supplementation elicits is in K^+^. Nonetheless, shifts in Cl^−^ similar to those observed in the present study have been suggested to drive K^+^ back to the muscle fiber through inward rectifier channels, which assist in returning the cell back to resting membrane potential (32). A well-designed study by Bouclin et al. (14) also showed that when an increased K^+^ and reduced Na^+^ were altered in combination, the effects on twitch and tetanic contractions were greater than the changes in these ions in isolation. It is more likely therefore, that collective changes in electrolyte regulation explain the ergogenic mechanism of NaHCO_3_ supplementation. Further research should therefore continue to explore the effects of NaHCO_3_ supplementation on the SID and exercise performance.

One individual presented moderate to high GI discomfort following NaHCO_3_ ingestion and displayed an ergolytic effect on performance. These findings agree with prior investigations suggesting GI discomfort might be a factor that negates the performance improvement from NaHCO_3_ (33–35). Indeed, Saunders et al. (33) reported upon removing participants who suffered GI discomfort following NaHCO_3_ ingestion, only then did total work done (TWD) improve (*p* = 0.01, *d* = 0.25) compared to when all participants were included (*p* = 0.16, *d* = 0.14). However, performance benefits in combination with the onset of GI discomfort have occurred previously, whilst there is a lack of a direct link between GI discomfort and exercise performance following NaHCO_3_ ingestion (20, 36). Individuals that suffer from severe GI discomfort could benefit from a lower dose of NaHCO_3_, as 0.2 g.kg^−1^ BM NaHCO_3_ has been shown to produce similar ergogenic responses whilst significantly reducing GI discomfort (36). Alternatively, the athlete could consider gastric bypass methods of delivery (i.e., enteric coated capsules), as novel data has suggested this may be suitable to reduce GI discomfort but still achieve the required increase in acid base balance (37, 38); although the performance responses are currently unclear. Further research should explore both lower doses of NaHCO_3_ and the use of gastric bypass methods of delivery to understand the link between GI discomfort and performance following NaHCO_3_ ingestion.

A limitation of this study is the small sample size, meaning further work is required to establish the impact of manipulating post-exercise acid base balance on performance and recovery. Despite this, the participant cohort were of an elite standard which are typically difficult to access. The current study findings therefore still have high practical application in sports performance, although further research with larger sample sizes are required. These findings compliment previous research investigating NaHCO_3_ supplementation and exercise performance within lesser-trained combat athletes (9, 24, 39) and support the use of NaHCO_3_ supplementation to promote superior recovery.

## Conclusion

The use of NaHCO_3_ is a suitable ergogenic aid to achieve a greater magnitude of acid base balance recovery and improve subsequent boxing performance within elite level boxers. Being the first study to assess this within an elite participant cohort, the results of this study are of significance to athletes and coaches in an applied setting. Boxers within the elite category could therefore implement this strategy to augment training performance and potentially the subsequent adaptations. One participant did present ergolytic effects following NaHCO_3_ ingestion however, which seemed to be due to high GI discomfort. Athletes should therefore trial NaHCO_3_ ingestion to assess individual tolerability. Future research should implement similar recovery interventions within a larger sample of elite athletes to explore the effectiveness of NaHCO_3_ supplementation as a recovery strategy.

## Data Availability Statement

The datasets generated for this study are available on request to the corresponding author.

## Ethics Statement

The study received institutional ethics committee approval (University of Derby, UK) prior to any testing, and participants were informed of the details of the study, both verbally and in writing, prior to providing written informed consent in accordance with the Declaration of Helsinki.

## Author Contributions

This study was conceived by MH and designed by MH and SR. Data were collected by SR and analyzed by LG and MH. Data interpretation and manuscript preparation were undertaken by LG, MH, LM, and SS. All authors approved the final version of the paper.

### Conflict of Interest

The authors declare that the research was conducted in the absence of any commercial or financial relationships that could be construed as a potential conflict of interest.
